# Incremental and decremental cardiopulmonary exercise testing protocols produce similar maximum oxygen uptake in athletes

**DOI:** 10.1038/s41598-021-92191-2

**Published:** 2021-06-23

**Authors:** Nuno Manuel Frade de Sousa, Danilo Rodrigues Bertucci, Gabriel Medeiros de Sant’Ana, Pedro Luiz Ribeiro Angelucci Padua, Diogo Mello da Rosa

**Affiliations:** 1 Faculdade Estácio de Sá, Laboratory of Exercise Physiology, Department of Physical Education, Av Armando Duarte Rabello 194/705, Vitória, ES 29092-280 Brazil; 2 Institute of Biosciences and Postgraduate Program in Movement Sciences, Universidade Estadual Paulista Júlio de Mesquita Filho (UNESP), Rio Claro, SP Brazil

**Keywords:** Physiology, Circulation, Respiration

## Abstract

The aim of the study was to evaluate and compare the maximal oxygen uptake ($\dot{\mathrm{V}}$O_2max_) achieved during incremental and decremental protocols in highly trained athletes. Nineteen moderate trained runners and rowers completed, on separate days, (i) an initial incremental $\dot{\mathrm{V}}$O_2max_ test (INC) on a treadmill, followed by a verification phase (VER); (ii) a familiarization of a decremental test (DEC); (iii) a tailored DEC; (iv) a test with decremental and incremental phases (DEC-INC); (v) and a repeated incremental test (INC_F_). During each test $\dot{\mathrm{V}}$O_2_, carbon dioxide production, ventilation, heart and breath rates and ratings of perceived exertion were measured. No differences were observed in $\dot{\mathrm{V}}$O_2max_ between INC (61.3 ± 5.2 ml kg^−1^ min^−1^) and DEC (61.1 ± 5.1 ml kg^−1^ min^−1^; average difference of ~ 11.58 ml min^−1^; *p* = 0.831), between INC and DEC-INC (60.9 ± 5.3 ml kg^−1^ min^−1^; average difference of ~ 4.8 ml min^−1^; *p* = 0.942) or between INC and INC_F_ (60.7 ± 4.4 ml kg^−1^ min^−1^; *p* = 0.394). $\dot{\mathrm{V}}$O_2max_ during VER (59.8 ± 5.1 ml kg^−1^ min^−1^) was 1.50 ± 2.20 ml kg^−1^ min^−1^ lower (~ 2.45%; *p* = 0.008) compared with values measured during INC. The typical error in the test-to-test changes for evaluating $\dot{\mathrm{V}}$O_2max_ over the five tests was 2.4 ml kg^−1^ min^−1^ (95% CI 1.4–3.4 ml kg^−1^ min^−1^). Decremental tests do not elicit higher $\dot{\mathrm{V}}$O_2max_ than incremental tests in trained runners and rowers, suggesting that a plateau in $\dot{\mathrm{V}}$O_2_ during the classic incremental and verification tests represents the maximum ceiling of aerobic power.

## Introduction

The concept of maximal oxygen uptake ($\dot{\mathrm{V}}$O_2max_) was introduced in 1924 after the works of the Nobel laureate Archibald Hill and his colleagues^1^. They observed a linear relationship between workload and oxygen uptake until the $\dot{\mathrm{V}}$O_2max_ was reached and proposed that the body has a limited capacity to uptake, transport and/or consumption the oxygen, called physiological ceiling for cardiorespiratory fitness^1^. Since then, $\dot{\mathrm{V}}$O_2max_ has been considered one of the most important indicators of endurance capacity^2^ and its determination has become one of the most widely used test procedures in experimental and clinical exercise physiology^3^ for testing the cardiorespiratory fitness and performance of athletes, the efficacy of training strategies or ergogenic aids and for quantifying the functional predations of chronic diseases^3,4^.

Because $\dot{\mathrm{V}}$O_2max_ is an important outcome for both physical performance and health status, test designs that increase the reliability and validity of $\dot{\mathrm{V}}$O_2max_ determination have widespread applicability. Since the 1970s, the maximal incremental exercise test has become a popular method for establishing $\dot{\mathrm{V}}$O_2max_^5^. The $\dot{\mathrm{V}}$ O_2max_ determination typically requires subjects to continue the incremental exercise test until they reach their limit of tolerance^6^. However, Bentley, et al.^7^ draw attention to methodological factors that influence physiological parameters such as $\dot{\mathrm{V}}$O_2max_ in trained endurance athletes during the incremental exercise protocol. In this sense, the application of criteria to assess whether a ‘true’ $\dot{\mathrm{V}}$ O_2max_ was achieved during an incremental test is still widely discussed by exercise physiologists.

The first and primary criterion traditionally used for establishing that a ‘true’ $\dot{\mathrm{V}}$O_2max_ has been reached is when there is no increase in $\dot{\mathrm{V}}$O_2_ in response to an increase in work rate at the end of the incremental test: a plateau in $\dot{\mathrm{V}}$O_2max_^8–11^. Unfortunately, the $\dot{\mathrm{V}}$O_2max_ plateau is not always identified in all test individuals^12,13^. Age, modality tested, data processing, physical fitness and test design can influence the incidence of plateaus in $\dot{\mathrm{V}}$ O_2max_^9,14,15^. In those instances where a $\dot{\mathrm{V}}$O plateau is not attained as definitive evidence of $\dot{\mathrm{V}}$O_2max_, investigators commonly elect to substantiate that $\dot{\mathrm{V}}$O_2max_ was actually achieved by utilization of secondary criteria assumed to validate $\dot{\mathrm{V}}$O_2max_—heart rate (HR) ≤ 5% of the age-predicted (220-age) maximum, blood lactate concentration ≥ 8 mM, or respiratory exchange ratio (RER) > 1.00, 1.10, or 1.15^16^. Utilization of these criteria can allow for a 30–40% underestimation of the ‘true’ $\dot{\mathrm{V}}$O_2max_ and/or an errant rejection of tests in which subjects had actually achieved their $\dot{\mathrm{V}}$O_2max_^16^. To reduce these limitations, a supramaximal verification test, first proposed by Thoden^17^ was used in this century to confirm the ‘true’ $\dot{\mathrm{V}}$O_2max_^16,18–21^. The supramaximal verification test involves a single square-wave bout of exercise performed shortly after the incremental test with a workload higher than the last completed stage of the maximal incremental test. The $\dot{\mathrm{V}}$O_2max_ results were compared between phases and consistent $\dot{\mathrm{V}}$O_2max_ values in the incremental and verification phases confirms that a ‘true’ $\dot{\mathrm{V}}$O_2max_ has been attained^10,19–22^.

Beyond the methodological factors, there also remains considerable debate regarding the factors regulating or limiting $\dot{\mathrm{V}}$O_2max_^23–25^. The classical model, based on the studies of Hill, et al.^1^ and in accordance with the ‘true’ $\dot{\mathrm{V}}$O_2max_ achieved during incremental and verification tests, proposed that $\dot{\mathrm{V}}$O_2max_ is limited by the maximal cardiac output (cardiac limitation)^26^ and the diffusional transport of oxygen out of the muscle microcirculation (muscle limitation)^27^. However, at the beginning of this century, Noakes and Marino^28^ introduced another theory into this discussion, which states that the cardiovascular system never reaches a limit of work and that $\dot{\mathrm{V}}$O_2max_ is regulated, rather than limited, by the number of motor units recruited in the exercising limbs, which is always submaximal. This model proposes that the central nervous system (a central governor) controls the circulation during severe exercise and that there is always both cardiovascular and neuromuscular reserve upon exhaustion during incremental exercise^23^; however, the brain stops the exercise to prevent catastrophic failure in the body system^28^.

Thus, the ‘central governor’ model, supported by the experimental design with decremental tests of Beltrami, et al.^29^ suggests that the $\dot{\mathrm{V}}$O_2max_ achieved during incremental exercise is not the ‘true’ $\dot{\mathrm{V}}$O_2max_. Beltrami, et al.^29^ supported the decremental test design with: (i) an incremental test may cause more anticipatory stress, which may lead to a difference in blood flow response; (ii) a decremental test, with workload progressively easier, might relax brain controls directing the termination of exercise^28,30^; (iii) evidence suggests that submaximal decremental protocols produce higher-than-expected $\dot{\mathrm{V}}$O_2_ values compared with a similar power output during an incremental protocol^31,32^. In fact, Beltrami, et al.^29^ showed that decremental protocols elicit significantly higher $\dot{\mathrm{V}}$O_2max_ values (~ 4%) than incremental protocol in subjects involved in running and cross-country skiing training. On the other hand, Taylor, et al.^33^, using similar decremental tests in running and triathlon training subjects, did not report differences in the $\dot{\mathrm{V}}$O_2max_ achieved during incremental or decremental tests, and both protocols elicited a similar cardiovascular response. These results showed that $\dot{\mathrm{V}}$O_2max_ determination is still challenging, not only from a methodological point of view but also from the factors limiting $\dot{\mathrm{V}}$O_2max_, although it is widely used and well established. Considering the scarce literature on decremental protocols for testing the regulatory factors of $\dot{\mathrm{V}}$O_2max_, the aim of this study was to evaluate and compare the $\dot{\mathrm{V}}$O_2max_ achieved during incremental and decremental protocols in trained athletes.

## Methods

### Participants

The study included 19 moderate trained men who competed in the junior category at a national or, in some cases, international level (15 distance runners and 4 rowers; age 17.4 ± 1.0 years, body mass 69.2 ± 6.3 kg, height 174.6 ± 4.6 cm and body fat 8.3 ± 1.6%) with at least 6 months of uninterrupted training. The rowers were former runners and they had running as part of their training routine. The participants were injury-free and did not use any controlled drugs or nutritional supplementation during the experimental protocols. All procedures were approved by the university´s Ethics Committee (51127515.8.0000.5284) and were conducted in accordance with the Declaration of Helsinki. The subjects were kept informed of experimental procedures and risks and signed informed consent before participation in the study. The study followed the STROBE (STrengthening the Reporting of OBservational studies in Epidemiology). All trial sessions took place in one laboratory using the same equipment.

### Experimental design

**Figure 1:**
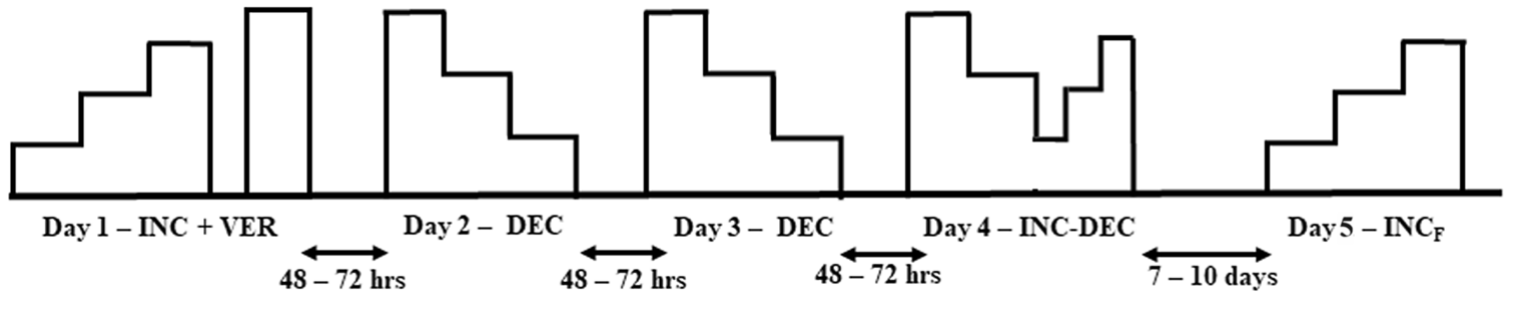
Experimental design. First four sessions were separated by 48–72 h between them and the fifth by 7–10 days. *INC* incremental test, *VER* verification phase test, *DEC* decremental test, *DEC–INC* decremental–incremental test, *INC*_*F*_ incremental final.

The participants visited the university laboratory on five occasions (Fig. 1) to complete the study. The sessions were separated by 48–72 h between the first four visits and by 7–10 days between the fourth and the fifth visit. All tests were performed using a motored treadmill (Super ATL, Inbrasport, Porto Alegre, RS, Brazil), a gas analyser (Metalyzer II, Cortex, Leipzig, Germany) that was calibrated before each test according to the manufacturer’s instructions, a heart rate monitor (Polar S810i, Kemple, Finland) and a Borg perception effort scale^34^.

On the first visit, the participants performed an incremental test (INC) until volitional exhaustion, and after 15 min a verification phase test (VER) was performed. A familiarization decremental test (DEC) was performed on the second visit and a tailored DEC on the third visit. On the fourth visit, a test with decremental and incremental phases (DEC-INC) was applied. Finally, a repeated incremental test was applied on the fifth visit (INC_F_).

The participants were instructed to avoid hard training sessions during the data collection period (15–20 days) and not to ingest caffeine or any kind of stimulant for 6 h before the tests. Tests were scheduled at the same time of the day (afternoon; between 3 and 5 p.m.) and laboratory conditions were stable for the duration of the study (ambient temperature 20–22 °C, relative air humidity 50–60%). All tests were performed on a motor-driven treadmill with a constant inclination of 5% (to ensure that participants do not exceed the capacity of the treadmill). All trial sessions were completed in 15–20 days by each participant. All tests were preceded by a 5-min warm-up at 10 km h^−1^ with no treadmill inclination and participants were verbally encouraged throughout each test.

### Incremental test (INC) and verification phase test (VER)

INC started at 9 km h^−1^ and 5% grade and the speed was increased by 1 km h^−1^ every minute until volitional exhaustion. Fifteen minutes after the end of INC in the first session the participants performed VER to confirm the values of $\dot{\mathrm{V}}$O_2max_. VER began at 10 km h^−1^ and 5% grade for 1 min and then the speed was increase to 1 km h^−1^ above the maximum speed reached in the previous INC. Participants were instructed to run at that speed for as long as they could. During the interval between INC and VER they were advised to rest or walk. The value of $\dot{\mathrm{V}}$O_2max_ was considered the highest after a mean of 20 s (with the data interpolated on a second by second basis and then averaged to retrieve the $\dot{\mathrm{V}}$O_2max_ value). INC was repeated on the fifth visit (INC_F_) without VER in order to mislead the training effect on $\dot{\mathrm{V}}$O_2max_ during the experimental protocol.

### Decremental test (DEC)

DEC protocols (familiarization and tailored) were established as a function of the result from the first session (INC + VER). The familiarization DEC was performed at the second visit. The first stage consisted of a graded increase in the speed of the treadmill until it reached that in VER (2 min at 9–10 km h^−1^, 1.5 min at 12–13 km h^−1^ and 30 s at 15.5–16.5 km h^−1^). This graded increase in speed was included to diminish the gap in speed between the warm-up and the high-intensity start of the test. After reaching the VER speed, participants ran for 60% of the time that each had managed during VER (usually around 1 min). After this stage, speed was decreased by 1 km h^−1^ and maintained for 30 s. The speed in the following stages had consecutive decreases of 0.5 km h^−1^ that were maintained for 30, 45, 60, 90 and 120 s, respectively. The tailored DEC was performed at the third visit, the duration of the stages being determined by the individual reaction of the participant to the familiarization DEC. Some adjustments (increase or decrease in stage duration) were made to ensure that DEC was longer than 5 min. The value of $\dot{\mathrm{V}}$O_2max_ was considered the highest after a mean of 20 s. No physiological data were measured during the familiarization DEC.

### Decremental and incremental test (DEC-INC)

DEC-INC was established as a function of the result from the DEC session. The first phase was similar to DEC and lasted until reaching the speed for $\dot{\mathrm{V}}$O_2max_ achieved during DEC. After reaching this speed, the second phase of DEC-INC consisted of consecutive 10 s stages with increments of 0.5 km h^−1^ until volitional exhaustion. The value of $\dot{\mathrm{V}}$O_2max_ was considered the highest after a mean of 20 s.

### Instruments and data handling

All physiological data were collected within a fixed time of 10 s, exported from the analyser software into Excel spreadsheets. During INC, data were collected until the end of the final completed stage. Data collected during VER, DEC and DEC-INC were considered up to the final time (at least 30 s) the respective test had been collected. This was done to ensure that comparison between INC and VER was done at two different workloads, as required to define a plateau in $\dot{\mathrm{V}}$O_2_. The maximum test confirmation criteria for INC and INC_F_ were: (a) the volitional exhaustion and a plateau in $\dot{\mathrm{V}}$O_2_; (b) when the plateau in $\dot{\mathrm{V}}$O_2_ was not achieved, the volitional exhaustion and one of the following criteria: heart rate (HR) ≤ 5% of the age-predicted (220-age) maximum or respiratory exchange ratio (RER) > 1.00. A plateau in $\dot{\mathrm{V}}$O_2_ during INC was accepted if the change in $\dot{\mathrm{V}}$O_2_ during the highest 30-s interval between the two final stages of the test was less than half of the normal stage-to-stage change in $\dot{\mathrm{V}}$O_2_ during the initial (linear) parts of the tests for each subject^29^. The average stage-to-stage difference in $\dot{\mathrm{V}}$O_2_ for all participants was calculated as 320 ± 66 ml min^−1^, so the plateau phenomenon was defined as a change in $\dot{\mathrm{V}}$O_2_ < 160 ± 33 ml min^−1^ (or an average of ± 2.2 ml kg^−1^ min^−1^, considering the average body mass of the participants) between the two final stages of the test. The same criterion was used to define a plateau in $\dot{\mathrm{V}}$O_2_ between the $\dot{\mathrm{V}}$O_2max_ values measured during INC and VER, because VER was performed at one stage higher than the maximal stage completed during INC^29^.

Considering the stage when $\dot{\mathrm{V}}$O_2max_ was reached for each test, minute ventilation (VE), breathing rate (BR), respiratory exchange ratio (RER), heart rate (HR) and rating of perceived exertion (RPE) were evaluated.

### Statistical analysis

Results are expressed as the mean ± standard deviation (SD). The normality of the data was tested and confirmed by Shapiro–Wilk’s test (*p* > 0.05), which allowed the use of parametric statistics. A one-way repeated measures ANOVA was used to compare the speed and respiratory and psychological variables at $\dot{\mathrm{V}}$O_2max_ between the proposed tests (INC, VER, DEC, DEC-INC and INC_F_). Compound sphericity was verified by the Mauchley test. When the assumption of sphericity was not met, the significance of F-ratios was adjusted according to the Greenhouse–Geisser procedure. Tukey’s post-hoc test with the Bonferroni adjustment was applied in the event of significance. Sample size was determined using G*Power version 3.1.3^35^, based on the difference in $\dot{\mathrm{V}}$O_2max_ between INC and VER during a pilot study with *n* = 5 and the $\dot{\mathrm{V}}$O_2max_ presented by Beltrami, et al.^29^. Considering the effect size (ES) achieved (~ 0.75), alpha error of 0.05 and power (1 − β) of 0.80, the required sample size was *n* = 16. All analyses were conducted using the Statistical Package for Social Science software, version 21.0 (SPSS Inc., Chicago, IL, USA), and the significance level was set at *p* < 0.05. The typical error in the test-to-test changes in $\dot{\mathrm{V}}$O_2max_ was calculated using an Excel spreadsheet that calculates reliability statistics for consecutive pairs of trial sessions^36^.

### Ethics approval

National Research Ethics Committee Brazil (CAEE 51127515.8.0000.5284).

## Results

**Figure 2:**
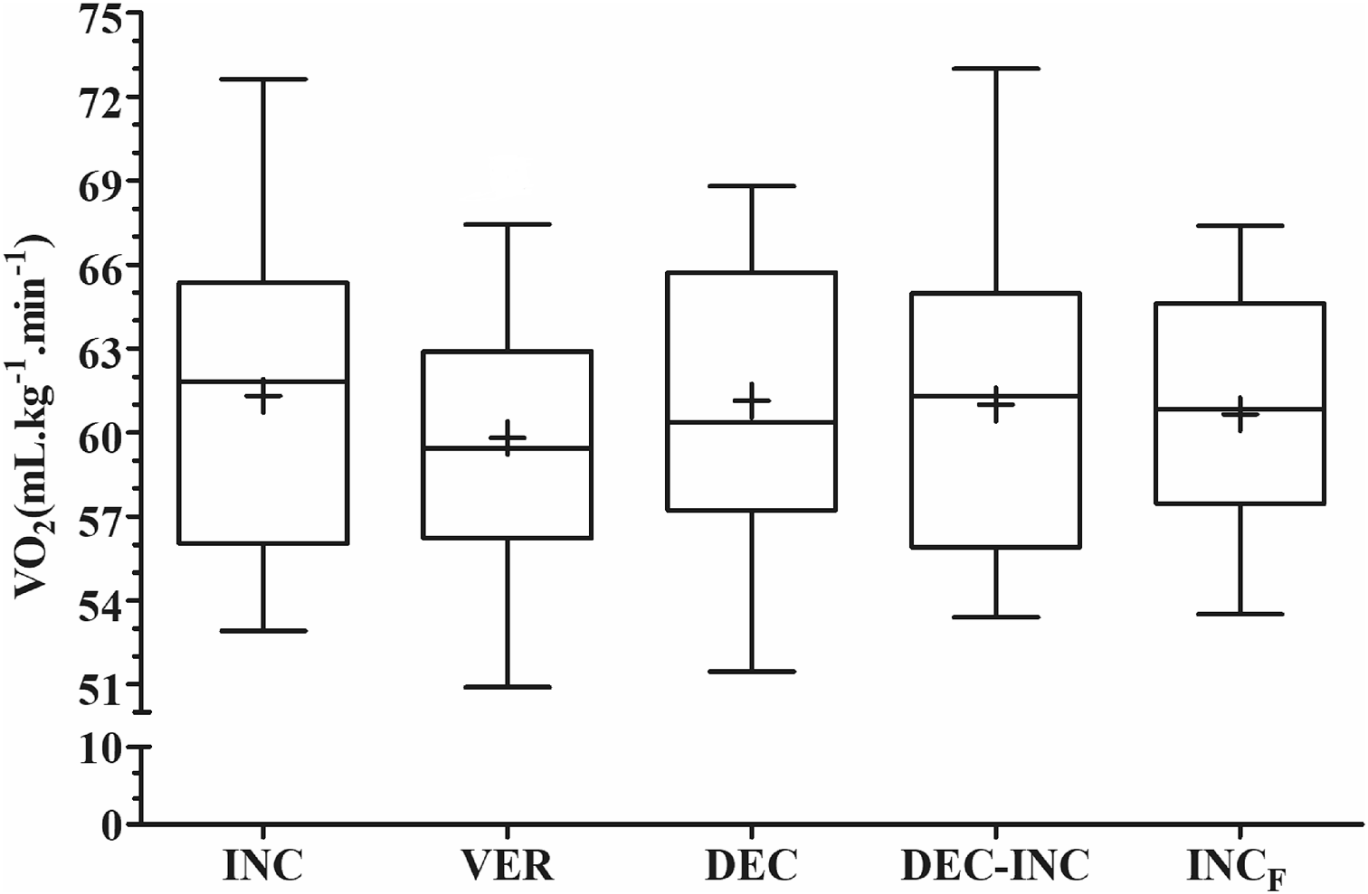
Maximal oxygen consumption ($\dot{\mathrm{V}}$O_2max_) during the five different protocols. *INC* incremental test, *VER* verification phase test, *DEC* decremental test, *DEC–INC* decremental–incremental test, *INC*_*F*_ final incremental test. No statistically significant differences were observed between protocols.

The plateau phenomenon in $\dot{\mathrm{V}}$O_2_ was observed in 13 participants during INC, 7 participants during VER and 12 participants during INC_F_. When the INC and VER protocols were combined, 17 participants achieved the plateau phenomenon. Figure 2 shows the $\dot{\mathrm{V}}$O_2max_ achieved during all five protocols. The protocol intervention did not elicited statistically significant changes in $\dot{\mathrm{V}}$ O_2max_ values (*F*(60, 4) = 0.80, *p* = 0.528, ES = 0.05. Post hoc analysis with a Bonferroni adjustment revealed that there were no differences in $\dot{\mathrm{V}}$O_2max_ between INC and INC_F_ (61.3 ± 5.2 vs. 60.7 ± 4.4 ml kg^−1^ min^−1^, respectively; *p* = 1.000), indicating no familiarization between tests. No differences in $\dot{\mathrm{V}}$O_2max_ were observed with post hoc analysis between INC and VER (59.8 ± 5.1 ml kg^−1^ min^−1^; average difference of 1.50 ± 2.20 ml kg^−1^ min^−1^, ~ 2.45%; *p* = 0.127), INC and DEC (61.1 ± 5.1 ml kg^−1^ min^−1^; average difference of 0.17 ± 3.30 ml kg^−1^ min^−1^, ~ 11.58 ml min^−1^; *p* = 0.831) or between INC and DEC-INC (60.9 ± 5.3 ml kg^−1^ min^−1^; average difference of ~ 0.07 ± 4.20 ml kg^−1^ min^−1^, 4.8 ml min^−1^; *p* = 0.942), which is 73% lower than the threshold for a $\dot{\mathrm{V}}$O_2_ plateau during INC (2.2 ml kg^−1^ min^−1^, 159.9 ml min^−1^). Considering these protocols from the same objective, to evaluate $\dot{\mathrm{V}}$O_2max_, the typical error in test-to-test changes for $\dot{\mathrm{V}}$O_2max_ over the five tests was 2.4 ml kg^−1^ min^−1^ (95% CI = 1.4–3.4 ml kg^−1^ min^−1^).

All physiological variables evaluated during the protocols are listed in Table 1. The protocol intervention did not elicited statistically significant changes in VE [(*F*(60, 4) = 2.05, *p* = 0.099, ES = 0.12] and RPE [(*F*(36, 3) = 2.92, *p* = 0.057, ES = 0.20] values. Statistically significant changes were observed in BR [(*F*(60, 4) = 4.00, *p* = 0.006, ES = 0.21] and HR [(*F*(36, 3) = 10.05, *p* ≤ 0.0005, ES = 0.46], with lower BR values in INC_F_ than in INC and lower HR values in DEC and DEC-INC than in INC and INC_F_. The protocol intervention elicited statistically significant changes in RER values [(*F*(60, 4) = 19.44, *p* ≤ 0.0005, ES = 0.56], with the lower RER achieved during VER and the highest RER during DEC and DEC-INC. The INC and INC_F_ did not present statistically significant differences in RER. The protocol also elicited statistically significant changes in v $\dot{\mathrm{V}}$O_2max_ [(*F*(60, 4) = 9.20, *p* ≤ 0.0005, ES = 0.38] and TTE [(*F*(60, 4) = 53,78 *p* ≤ 0.0005, ES = 0.78], with the VER protocol presented higher v$\dot{\mathrm{V}}$O_2max_ and lower TTE than all other protocols. The DEC protocol also presented lower TTF than INC and INC_F_.

**Table 1:** Physiological variables achieved at $\dot{\mathrm{V}}$O_2max_ during the different protocols.

	INC	VER	DEC	DEC–INC	INC_F_
$\dot{\mathrm{V}}$O_2max_ (L min^−1^)	4.23 ± 0.58	4.13 ± 0.57	4.22 ± 0.55	4.23 ± 0.55	4.15 ± 0.43
VE (L min^−1^)	119.9 ± 14.7	118.7 ± 14.9	116.6 ± 12.9	116.7 ± 14.4	111.8 ± 12.1
BR (BR min^−1^)	64.6 ± 6.1	65.9 ± 7.3	61.0 ± 7.1	61.9 ± 7.4	59.6 ± 6.9^†^
RER	1.09 ± 0.05	1.00 ± 0.07*	1.14 ± 0.03*^†^	1.16 ± 0.87*^†^	1.10 ± 0.05^†‡^
HR (b min^−1^)	197.3 ± 7.3	–	193.1 ± 5.7*	190.8 ± 8.0*	195.7 ± 8.0^‡§^
RPE	16.8 ± 2.2	–	14.8 ± 3.8	15.5 ± 4.0	17.2 ± 2.6
TTE (s)	477 ± 72	130 ± 24*	393 ± 43*^†^	403 ± 60^†^	477 ± 69^†‡^
v$\dot{\mathrm{V}}$O_2max_ (km h^−1^)	15.8 ± 1.1	16.8 ± 1.1*	15.7 ± 1.7^†^	16.0 ± 1.2^†^	16.0 ± 1.1^†^

$\dot{V}$*O*_*2max*_ maximal oxygen consumption, *VE* minute ventilation, *BR* breath rate, *RER* respiratory exchange rate, *HR* heart rate, *RPE* rating of perceived exertion, *TTE* time to exhaustion, *v*$\dot{V}$*O*_*2max*_ intensity at $\dot{\mathrm{V}}$O_2max_, *INC* incremental test, *VER* verification phase test, *DEC* decremental test, *DEC–INC* incremental test, *INC*_*F*_ final incremental test.

*p < 0.05 for INC; ^†^p < 0.05 for VER; ^‡^p < 0.05 for DEC; ^§^p < 0.05 for DEC-INC; – did not recorded.

**Figure 3:**
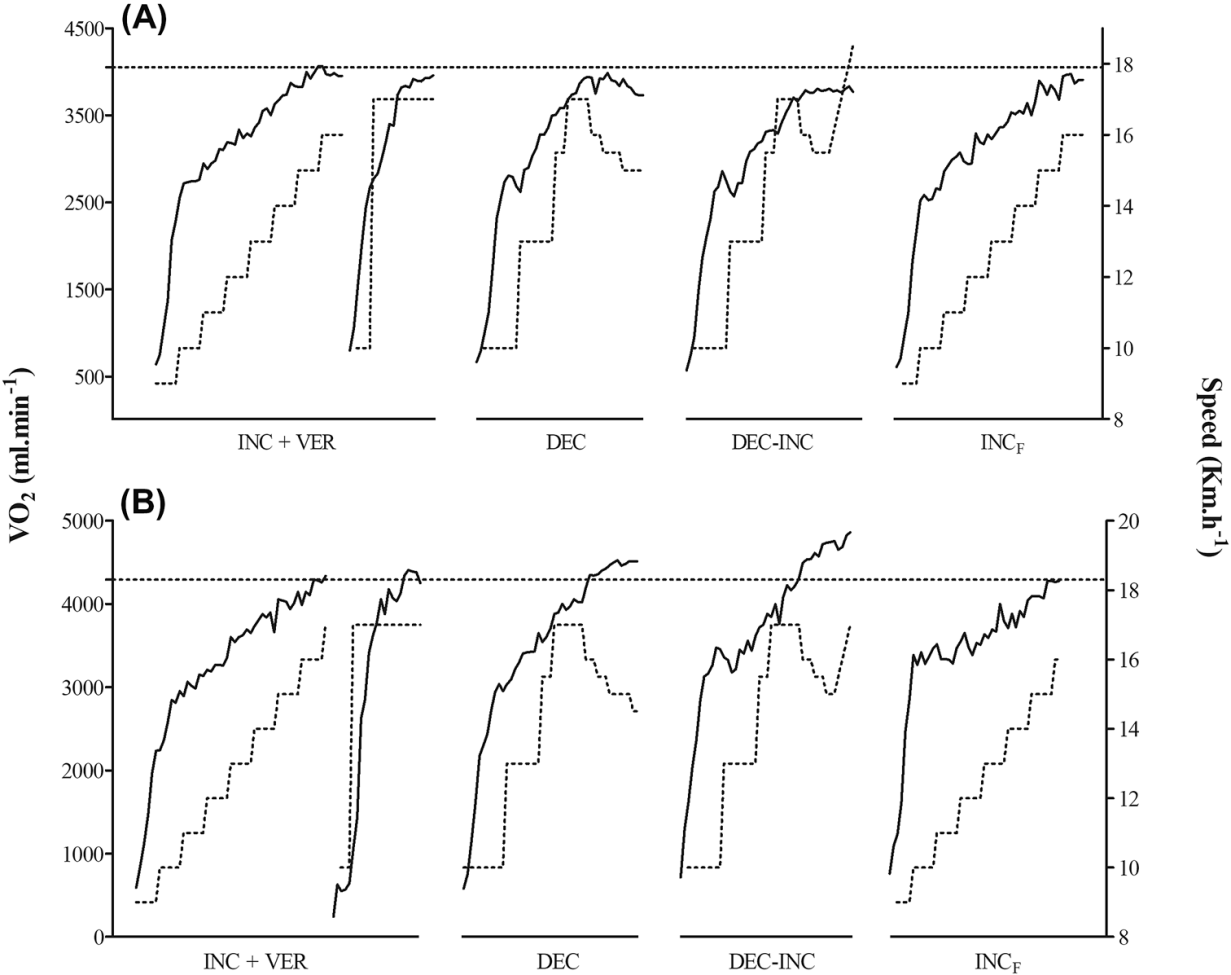
Maximal oxygen consumption ($\dot{\mathrm{V}}$O_2max_) and speed treadmill during the five different protocols for a participant who presented similar values of $\dot{\mathrm{V}}$O_2max_ between INC and DEC (**A**) and a participant who presented very different values of $\dot{\mathrm{V}}$O_2max_ between INC and DEC (**B**).

A graphical illustration of the relationship between $\dot{\mathrm{V}}$O_2_ and speed in the different trial sessions for two volunteers is presented in Fig. 3: (A) a participant who presented very similar values of $\dot{\mathrm{V}}$O_2max_ between protocols; and (B) a participant who presented different values of $\dot{\mathrm{V}}$O_2max_ between INC and DEC or DEC-INC. When an individual analysis of the sampled subjects was performed, only six achieved a $\dot{\mathrm{V}}$O_2max_ during DEC and DEC-INC (three are the same subjects) that was 160 ± 33 ml min^−1^ (plateau phenomenon calculated in INC) greater than the $\dot{\mathrm{V}}$O_2max_ achieved during INC. None of the subjects presented a $\dot{\mathrm{V}}$O_2max_ during VER that was 160 ± 33 ml min^−1^ greater than the $\dot{\mathrm{V}}$O_2max_ achieved during INC. Of the sample that did not develop the plateau phenomenon (six subjects), only two achieved a $\dot{\mathrm{V}}$O_2max_ during DEC and DEC-INC (the same subjects) that was 160 ± 33 ml min^−1^ greater than the $\dot{\mathrm{V}}$O_2max_ achieved during INC. However, no difference in $\dot{\mathrm{V}}$O_2max_ was observed between INC and DEC when only the six subjects were evaluated (*n* = 6; INC = 61.4 ± 4.8 ml kg^−1^ min^−1^; DEC = 62.9 ± 5.0 ml kg^−1^ min^−1^; *p* = 0.160). Individual $\dot{\mathrm{V}}$O_2max_ response results can be consulted in the Supplementary Information.

## Discussion

The results of the present study showed that, regardless of the test performed, the $\dot{\mathrm{V}}$O_2max_ achieved in each test was very similar and its difference was smaller than the value of the plateau phenomenon between the last two stages of INC or even the measurement error in test-to-test changes. That is, contrary to the study by Beltrami, et al.^29^ no significant differences were found regarding the $\dot{\mathrm{V}}$O_2max_ values between INC and DEC. Moreover, the test proposed by us with decremental and incremental stages (DEC-INC) also showed no difference from INC. Thus, although more recently the theory of the plateau phenomenon has been criticized^37^, the results of the present study show that the plateau obtained at the time of exhaustion during a traditional test (i.e. INC) may infer the maximum ceiling of cardiorespiratory capacity and thus remain in exercise, as proposed by the classical theory^1,38,39^.

In an attempt to elucidate the validity of a cardiopulmonary test to assess maximal cardiorespiratory capacity, in addition to the plateau phenomenon, a series of validation criteria have been proposed in the literature, such as expected values of HR, RER and blood lactate concentrations^3^. In addition to those already cited, in order to improve the reliability of determining $\dot{\mathrm{V}}$O_2max_, VER has been proposed^18^. This test requires the participant to perform the exercise for as long as possible at a constant intensity above that achieved in INC. Therefore, even with increased intensity, if the $\dot{\mathrm{V}}$O_2max_ value is not higher than that found during INC it is assumed that the maximum aerobic power ceiling was reached during both tests^10,40,41^. From this application, the frequency with which the plateau was identified increased, strengthening the theory that discusses this phenomenon^41^ (when the INC and VER protocols were combined, 17 of the 19 participants achieved the plateau phenomenon.

Evidence using VER as an INC validity criterion has controversial results. At some point VER will identify different responses to INC depending on a series of factors (participants, tests performed, equipment used, etc.). Murias, et al.^42^ used VER (105%) to analyse the effectiveness of INC on a ramp format in young and old individuals. No significant differences were found between the two tests in the two populations, thus, although VER may be very seductive in finding different values, in that study it did not present any additional validation. Furthermore, Bhammar, et al.^43^ when comparing obese and non-obese children, found that the $\dot{\mathrm{V}}$O_2max_ values in VER (105% of v$\dot{\mathrm{V}}$O_2max_ during INC) were higher by approximately 6% and 10%, respectively. Barker, et al.^44^ also found that supramaximal testing at 105% of the power output achieved during ramp exercise did not increase the $\dot{\mathrm{V}}$O_2max_ achieved compared to the ramp test, thus suggesting the achievement of a true $\dot{\mathrm{V}}$O_2max_ during the initial ramp test for young people. However, the authors also concluded that the adherence to commonly used secondary criteria to validate a maximal effort (expected values of HR, RER and blood lactate concentration) in young people can result in either a submaximal $\dot{\mathrm{V}}$O_2max_ or a rejection of a participant’s $\dot{\mathrm{V}}$O_2max_ score despite a plateau being evident. Finally, Beltrami, et al.^29^ applied VER (110% of v$\dot{\mathrm{V}}$O_2max_ during INC) to highly trained individuals ($\dot{\mathrm{V}}$O_2max_ = 61.3 ml kg^−1^ min^−1^) and also found no significant differences, with very small variations between INC and VER. In the present study, the $\dot{\mathrm{V}}$O_2max_ during VER (110% of v$\dot{\mathrm{V}}$O_2max_ during INC) were not significantly different than during INC ($\dot{\mathrm{V}}$O_2max_ values during VER were 3% lower; p = 0.127; clinically irrelevant). It is clear that during INC the participants had reached the maximum ceiling of cardiorespiratory fitness, which was confirmed by the VER protocol. It is important to highlight that of the 6 participants that did not reach the plateau in $\dot{\mathrm{V}}$O_2max_ values during the INC, 3 reached during the VER protocol. These data sustained, once again, that the $\dot{\mathrm{V}}$O_2max_ achieved during INC is the maximum ceiling of cardiorespiratory fitness.

DEC was used by Beltrami, et al.^29^ to break with the traditional tests for determination of aerobic power and to evaluate the validity of the traditional INC in determining the maximum ceiling of cardiorespiratory fitness. Beltrami, et al.^29^ demonstrated that in DEC, their participants achieved significantly higher $\dot{\mathrm{V}}$O_2max_ values (4.4%) than those found when applying the traditional INC. Although the study was cited as being innovative as a function of protocol and approach, previous studies, even without the use of DEC as a tool for determining $\dot{\mathrm{V}}$O_2max_, are essential for understanding the phenomenon behind the oxygen uptake response to this stimulus^31,33,45–47^. The results of the present study show no differences between INC and DEC in the $\dot{\mathrm{V}}$O_2max_ achieved, or in the test developed by our research group, DEC-INC, which sought an increase in $\dot{\mathrm{V}}$O_2max_ with increasing oxygen uptake intensity after its decrease. We did not discard methodological differences between our study and the research of Beltrami, et al.^29^, mainly the difference in the gas analyzer and the difference between the procedures for collection and analysis of respiratory data. These results are in agreement with those obtained by Taylor, et al.^33^, who briefly compared the values of $\dot{\mathrm{V}}$O_2max_, cardiac output and systolic volume obtained by means of INC and DEC in very well-trained triathletes and runners, a population similar to that of the present study and also of Beltrami, et al.^29^. Taylor, et al.^33^ also found no significant differences between INC and DEC in maximal oxygen uptake values (57.29 ± 8.94 ml kg^−1^ min^−1^ vs. 60.82 ± 8.49 ml kg^−1^ min^−1^, respectively) or in cardiocirculatory values. They also concluded that DEC was not capable of causing higher values of maximal oxygen uptake but may be an alternative for this population. We do not share the same view, as DEC exhibits the same possible obstacles as INC (i.e. the need to establish initial loads, the duration of stages and the decrease in intensity) and is an ‘open-ended’ test^48^. In addition, due to the need to achieve high intensities in order to decrease intensity later, the error in high intensity selection is much higher than the error in intensity selection for INC. Thus, we have not assumed DEC to be a good alternative for determining $\dot{\mathrm{V}}$O_2max_, although it may be explored in the future.

Even with the emergence and acceptance of VER as a tool to try to validate the maximum exercise condition maintained by an individual, the debate about other factors that permeate the phenomenon of $\dot{\mathrm{V}}$O_2max_ remains intense. Among the most common theory is that the cardiovascular system limits $\dot{\mathrm{V}}$O_2max_ in a maximal or supramaximal situation^23,49^. According to Elliott, et al.^23^ the evidence is controversial. For instance, some studies show that cardiac output stabilizes at maximal and supramaximal loads, whereas others found that cardiac output does not stabilize in athletes. Barker et al.^44^ demonstrated in young people that the changes in the components of the Fick equation, that is maximal cardiac output and O_2_ extraction, were similar during ramp and supramaximal exercise, further supporting the notion that a true $\dot{\mathrm{V}}$O_2max_ was recorded during the ramp test. However, there is still little evidence related to the cardiovascular system in DEC. Taylor, et al.^33^ demonstrated no differences in maximal cardiac output and maximal systolic volume between INC and DEC, despite a tendency for higher values in INC and lower HR values in DEC. Interestingly, in the present study, the $\dot{\mathrm{V}}$O_2max_ achieved in DEC was obtained at intensities similar to those in INC (already in the descending phase of the test) but with lower TTE and lower values of HR and RER, demonstrating that there could still be a cardiorespiratory reserve but it was not translated into higher values of $\dot{\mathrm{V}}$O_2max_. Another front explored is the convection property of O_2_ in tissues, as well as its extraction of blood from the muscles^50^.

We cannot fail to mentioned that the results for identifying or not the ceiling effect found by different studies can be partially explained by methodological differences (e.g.; chronological age, sex, training level, training status, type of exercise, sports modality, ergometer, criteria for interruption and confirmation of the maximum test, criterion for plateau, motivation strategy, exercise protocol, $\dot{\mathrm{V}}$O_2_ sampling frequency, statistical analysis). Studies that used breath by breath analysis have a greater dispersion of $\dot{\mathrm{V}}$O_2_ data, which makes it difficult to identify a plateau. On the contrary, data collected every 10 s and analyzed in an average of 30 s (as in our study) decrease the variability of $\dot{\mathrm{V}}$O_2_ data, facilitating the identification of a plateau. On the other hand, there are few studies that make clear the criteria for interrupting the maximum test, as well as the criteria used to confirm the maximum test. This can really generate discrepant results and must be taken into consideration when comparisons are made.

According to Hill's classical theory (1924), running and rowing athletes reached the maximum ceiling of aerobic power by conventional testing (similar $\dot{\mathrm{V}}$O_2max_ in INC, VER, DEC, DEC-INC and INC_F_), in contrast to other studies^23,29^, that the $\dot{\mathrm{V}}$O_2max_ reached during conventional tests was lower than unconventional decremental tests. It is important to highlight that these results were achieved by trained athletes, with the possibility of different results in untrained individuals, since the participation of the different mechanisms behind the onset of fatigue/exhaustion is different depending on the training state. According to this study, the verification phase after the incremental protocol for athletes it’s not necessary; however, since not all athletes reach the $\dot{\mathrm{V}}$O_2max_ plateau during the incremental protocol, we recommend the verification phase when the excellence is sought (the chances that all athletes reach the plateau in $\dot{\mathrm{V}}$O_2max_ increases considerably when INC and VER are combined. Future research should aim to design a DEC protocol for non-athletic populations.

In summary, decremental tests do not elicit higher $\dot{\mathrm{V}}$O_2max_ than incremental tests in trained runners and rowers, suggesting that a plateau in $\dot{\mathrm{V}}$O_2_ during the classic incremental and verification tests represents the maximum ceiling of aerobic power.

## Supplementary Information

Supplementary Information.

## Data Availability

The views expressed in this publication are those of the author(s) and not necessarily those of the funders. All authors had full access to all of the data (including statistical reports and tables) in the study and can take responsibility for the integrity of the data and the accuracy of the data analysis. *Data sharing statement* Data are not publicly available, but applications for data sharing can be made. For enquiries, please contact NMFS (nunosfrade@gmail.com).
