# Supplementary material for: Incremental and decremental cardiopulmonary exercise testing protocols produce similar maximum oxygen uptake in athletes
Source: Sci Rep. 2021 Jun 23;11:13118. doi: 10.1038/s41598-021-92191-2 (PMC8222247; doi:10.1038/s41598-021-92191-2)
Supplement: Supplementary file 1 — Supplementary Information. [file 41598_2021_92191_MOESM1_ESM.pdf]

SUPPLEMENTARY INFORMATION

Incremental and decremental cardiopulmonary exercise testing protocols produce similar maximum oxygen uptake in athletes

Nuno Manuel Frade de Sousa (ORCID: 0000-0001-5854-616X)[1], Danilo Rodrigues Bertucci (ORCID: 0000-0002-9833-2390)[2], Gabriel Medeiros de Sant'Ana[1], Pedro Luiz Ribeiro Angelucci Padua[1], Diogo Mello da Rosa[1]

[1]Faculdade Estácio de Sá, Laboratory of Exercise Physiology, Department of Physical Education, Vitoria, ES, Brazil. [2]Universidade Estadual Paulista Júlio de Mesquita Filho (UNESP), Institute of Biosciences and Postgraduate Program in Movement Sciences, Rio Claro, SP, Brazil.

	VO2max(#1)	VO2max(#2)	VO2max(#3)	VO2max(#4)	VO2max(#5)
A	59,93	59,13	57,23	61,53	62,9
B	65,43	66,97	68,8	73	65,13
C	64,37	60,33	58,1	63,37	66,3
D	62,77	59,27	60,37	65,97	61,97
E	56,03	51,3	56,93	59,36	53,5
F	59,87	57,7	63,93	56,17	64,63
G	61,67	59,43	65,73	65,17	64,6
H	52,9	53,34	54,27	55,07	
I	65,17	66,8	68,27	64,9	60,7
J	61,83	61,67	61,7	59,5	60,97
K	55,7	56,23	55,8	53,7	55,23
L	53,77	50,9	51,46	53,4	57,56
M	65,63	62,83	66,7		
N	54,8	55,3	57,63	54,5	54,03
O	57,3	57,16	58,4	61,1	57,43
P	62,83	62,90	65,23	65,4	
Q	65,36	60,83	63,2	63,9	58,6
R	72,63	67,43	67,93	64,7	67,4
S	66,8	66,76	59,93	57,1	59,63

WEIGTH	VO2max(#1)	VO2max(#2)	VO2max(#3)	VO2max(#4)	VO2max(#5)
62,8	3,76	3,71	3,59	3,86	3,95
65,6	4,29	4,39	4,51	4,79	4,27
70,5	4,54	4,25	4,10	4,47	4,67
68,6	4,31	4,07	4,14	4,53	4,25
60,3	3,38	3,09	3,43	3,58	3,23
70,1	4,20	4,04	4,48	3,94	4,53
68	4,19	4,04	4,47	4,43	4,39
68,7	3,63	3,66	3,73	3,78	
69,5	4,53	4,64	4,74	4,51	4,22
63,7	3,94	3,93	3,93	3,79	3,88
71	3,95	3,99	3,96	3,81	3,92
64,3	3,46	3,27	3,31	3,43	3,70
61,9	4,06	3,89	4,13		
64,8	3,55	3,58	3,73	3,53	3,50
69,4	3,98	3,97	4,05	4,24	3,99
83,1	5,22	5,23	5,42	5,43	
82,6	5,40	5,02	5,22	5,28	4,84
67,7	4,92	4,57	4,60	4,38	4,56
75,9	5,07	5,07	4,55	4,33	4,53
